# The early interaction of the primary caregiver and the infant with respect to sex, gender and ego-identity

**DOI:** 10.3389/fpsyg.2026.1777617

**Published:** 2026-05-29

**Authors:** Gunnar Karlsson

**Affiliations:** Department of Education, Stockholm University, Sweden

**Keywords:** care, early interaction between primary caregiver and infant, ego-identity, gender, primary caregiver, psychoanalysis, sex

## Abstract

The sex/gender issue is a deeply controversial field of research. The intention of this article is limited to discussing the primary caregiver’s care of the infant with respect to sexual belongingness, gender ideals/character, as well as a relationship between I and You. A clarification of the relationship between sex, gender and ego-identity is presented. A section discussing the resistance to acknowledge preoedipal motherly care in psychoanalysis precedes an analysis of prenatal and postnatal care of the infant. The psychoanalysts Didier Anzieu, Donald W. Winnicott and Bracha Lichtenberg-Ettinger are the major theoreticians referred to when discussing prenatal and postnatal fetus/infant care. The last section of the article consists of a discussion of and some concluding remarks about: the refutation of essentialism concerning the sexual belongingness of the primary caregiver in the care of the infant; the value of openness with respect to sex and gender as well as drawing attention to the I-You relationship to deepen the understanding of beneficial conditions in infant care; the ill-fitness of phallic masculine gender ideals/character when it comes to infant care, and finally; under optimal conditions, the privileged position of the biological mother with respect to infant care.

## Introduction

The traditional psychoanalytic oedipal narrative of the child’s early upbringing consists of a mother–father-child unity. The primary caregiver is almost always taken to be the mother, or at least, a woman, whereas the secondary caregiver, entering into the infant’s upbringing later on, is assigned to the father or to a male person. The conceptualization of the mother–father-child entity has undergone changes in today’s Western world, as opposed to how it was understood in past psychoanalytic theory. By all means, there have been family forms other than the conventional nuclear family, such as single parents, female or male, same-sex parents, and lesbians or gay parent, in the past. However, what is new is the greater social recognition these forms of families have received in the Western world. These new family forms are investigated now as an important field of scientific research. Psychoanalytic literature on the topic is rare, although one can find a growing interest for LGBTQ+ questions within psychoanalytic research. See McNamara et al. (2025) for psychoanalysts “living outside the box” discuss parenting.^1^ Much of the research being conducted on the outcome of parents’ sexual belongingness for the development of the child (e.g., child gender role behavior, cognitive function, psychological adjustment, quality of parent–child relationship), is quantitative (for meta-analysis see, e.g., Suaréz et al., 2023; Zhang et al., 2023). Without questioning the value of such an approach, the typical psychoanalytic approach focuses upon unconscious processes. Some criticism has been launched against psychoanalysis for being too rigid in viewing parenthoods in terms of the nouns “mother” and “father,” which is said to run the risk of conveying a sexist view (cf., Taipale, in press).

The focus of this article is on the early interaction between the primary caregiver and the fetus/infant, most specifically on the issue of sex, gender and ego-identity. What is significant in the early interaction is the primary caregiver’s *care* of the infant for its continued emotional and psychological development. Several issues will be covered in this article: To begin with, I will briefly present my position concerning the sex/gender issue, including the description of an ego-identity. The ego-identity, which appears prior to the infant’s experience of sexual belongingness and gender ideals, is not usually taken into account in the field of sex/gender studies. This section is followed by a critique of patriarchal thinking found in psychoanalysis. A consequence of patriarchal thinking, most notable in the Oedipal theory can be understood as a defence against adequately acknowledging the human predicament of helplessness and dependence, and consequently, downplaying the phenomenon of care as well as an inadequate account of the mother as an object of identification for the infant. Thereafter, a section is devoted to the interaction between the mother and fetus in intra-uterine life. The psychoanalysts who are most important for this line of reasoning are Didier Anzieu, Donald W. Winnicott and Bracha Lichtenberg-Ettinger. The following section on the primary caregiver’s post-natal care are based on the writings of Anzieu and Winnicott. Generally, it is the mother who is conceived of as the most suitable for the care of the infant, although this is not without exception. In the ensuing section, in which sex, gender and the ego-identity are further elaborated, it is argued that the interaction between the primary caregiver and the infant should not be confined to sex and gender. It is also important to consider the importance of an I-You relationship, referring to Martin Buber’s philosophy, and to Mariam Alizade’s request to consider a “human dimension.” This article ends with a discussion in the form of four points, in which I draw conclusions concerning the early care of the infant from the perspectives of sex, gender and ego-identity.

## The field of sex/gender research

In the research field of sex and gender, it is obvious how difficult it has been to reach an agreement on how to define sex, gender and their possible relationship with one another. Two common pitfalls have been to either reduce gender to sex (biologism) or sex to gender (social constructionism). In the social sciences, during the last decades, the tendency has been to reduce sex to gender, more so than in psychoanalysis. This poststructuralist/social constructivist trend within the social sciences has been criticised for viewing sexual identities as shaped solely within language and the cultural sphere.^2^

Concerning the status of sex^3^ and gender in psychoanalysis, most psychoanalysts maintain a distinction between them, although different viewpoints can be discerned. As a matter of fact, Sigmund Freud was the first to substantially differentiate between sex and gender, according to Ethel S. Person and Lionel Ovesey. Since the German language only provided the concept “Geschlecht,” Freud did not have access to the terminological difference between sex and gender: “The insight that the existence of personality differences between the sexes required an explanation was a major intellectual leap, and it is Freud who must be credited with that insight. Thus, psychoanalysis was the first comprehensive personality theory that attempted to explain the origins of what we now call gender” (Person and Ovesey, 1983, p. 203).

However, in one of Nancy J. Chodorow’s later works, she abstains from differentiating between sex and gender: “I am calling the perspective […] the sexual difference perspective, though for me, it could equally be called the gender difference perspective” (2012a, p. 140). Such a position contrasts to Michael J. Diamond’s view, where gender becomes a construction anchored in biology and anatomy: “Rather than simply deconstructing gender dichotomies, I believe that sophisticated psychoanalytic theories must be able to sustain the necessary dialectical tension between traditional essentialist (either/or) thinking and a postmodern, constructivist (both/and) perspective” (2006, p. 1104).^4^

My solution to this terminological confusion is to conceive of one’s sex as experienced as something given, as something non-acquired.^5^ This sexual belongingness can be seen either from a first-person perspective or a third-person perspective. The first-person perspective is an inside perspective, that is, the experience of oneself as being a male, female or non-binary. This first-person perspective constitutes my sexual *identity.*^6^ A third-person perspective, on the other hand, is the intersubjective objectification of one’s body’s sexual belongingness.^7^ In other words, a person’s sexual identity, being female, male or non-binary, is not the outcome of choices and decisions. It is the notion of “gender” which involves actions, behavior, character and psychological and cultural levels of meaning. To avoid misunderstanding, the distinction between sex and gender is not to be understood as a body-psyche distinction. Gender is not a disembodied entity, but in the course of an individual’s development, the sexed body will be an articulation of gender characteristics. Elsewhere, I have argued that a possible striving for a phallic masculine (gender) identity is a failed project, in its denial of basic existential human conditions, such as vulnerability, helplessness and dependence (Karlsson, 2023). In the last section of this article, I will come back to the meaning of the phallic masculine project/character in the light of its unsuitability with regards to infant care.

Thus, one’s sexual identity is experienced as given, but needs to be consented to in order for it to not be experienced as a fault or ego-dystonic (i.e., an experienced incompatibility between one’s sense of oneself and one’s assigned sex). The fate of the transsexual is the experience that the sex assigned at birth does not align with one’s sexual identity.^8^ For example, a transsexual man’s sexual identity is being a man, although his sexual body, assigned at birth, does not correspond to his sexual identity as a man. For the transsexual, one’s sexual identity is not chosen, and not the outcome of possible behaviors or actions, but rather something one is, and always was, although this may not have been confirmed or realized by others (cf., Berlin and Shane, 2025; Ladin, 2025). In clinical work, one can discern the difference between sex and gender as the difference between, on the one hand, someone who seriously questions whether he really is a male, and on the other hand, someone who feels he has failed to behave as a male, such as asserting himself in a competitive situation, as a male is expected to do.

The relationship between sexual identity and gender is that gender is the individual’s interpretation of the sexually, culturally conditioned meaning (cf. Segal, 2007, p. 78). The individual is free to either comply to this meaning or realize the possibility of rejecting the idea that there is such a thing as a specific character attached to one’s sex (Karlsson, 2023). Gender can be conceived of as reified ideals and constructions based on cultural, social and historical ideas of what it means to belong to a specific sex, and thereby hiding both the openness to others and hampering one’s own freedom.^9^ It should be remembered that gender ideals are also shaped by intersectional relationships, such as race, ethnicity, sexuality, age, economic status, although here the focus is on gender, phallic masculinity.

To give a more nuanced picture of the field of sex/gender studies, I believe that the notion of “ego-identity” should be included. The constitution of human identity requires a conceptualization of a person that is not totally defined in terms of sex/gender. Alizade (2010, p. 199) expresses the importance of not restricting focus to sex and gender but paying attention to the simple fact that we are first and foremost human, something that she finds that psychoanalysis has not dealt with in sufficient depth. Labelling oneself in sex−/gender terms, presupposes an experience of an ego-identity, the earliest identity formation. To quote Alizade: “In the beginning, before having been classified as male or female, a human being is born” (2010, p. 203). In other words, there is a human dimension which “is tied to both pre-sexual and non-sexual questions based on mechanisms and defenses that spring from the interests of the ego and on the problems of the individual existence” (*Ibid.*, p. 205). These terminological distinctions between sex, gender and an ego-identity will come into play, when discussing their respective functions in the primary caregiver’s relationship to the fetus/infant.

## The patriarchal character of psychoanalytic theorizing

The phallic ideology has influenced psychoanalysis from its beginnings. This is seen firstly in the Oedipal theory. The master plot of Freudian theory is the oedipal conflict, which has been criticised for universalizing hierarchical social orders, demeaning women, and fostering sex-based hierarchies. The father is the main character in the Oedipal complex but is absent in the pre-oedipal sphere. The importance of the Oedipal complex for sexual identity has diminished in psychoanalytic thinking during the last few decades, by, among other things, the emergence of more relational thinking, and the highlighting of not only pre-oedipal mother–child processes, but also father-child interaction and triangulations such as mother–father-child.

The tendency in patriarchal societies to reserve the care of the young child to the mother risks a stereotyping and essentialising of maternal care. There is a risk of equating being a woman with being a mother (Leisse de Lustgarten, 2018) and creating a dichotomy for the mother between autonomy or mothering. Jessica Benjamin (1995) speaks about the importance for the mother as well as for the child, that the mother affirms her subjectivity, and that she has interests and desires for others outside the mother–child relationship. Layton describes that, in the 70s, the development of “[t]he ordinary contempt for mother postulated by Freud was replaced, in feminist theory, by an idealization of mothering and female biology” (2023, p. 134). The challenge is to navigate between Scylla, the idealization of the Oedipal father, and Charybdis, the idealization of the pre-oedipal mother. Both of these pitfalls imply a stereotyped, sometimes even an essentialist account of sexual belongingness.

For Freud, the Oedipal theory is centred around the castration complex, with the focus upon the question of whether one is equipped with a penis or not. In this article, I want to restrict my discussion to two aspects implied in allowing the Oedipal theory its central position in psychoanalysis. The first one to be dealt with is the downplaying of the importance of care that is crucial from the very beginning of a person’s life, even before the birth of the child. The other aspect is the inadequacy of phallic masculine ideals in terms of the early care of the infant.

Care is crucial in many ways for human beings, such as being able to take care of oneself, to develop caring relationships with others, and to have a responsible attitude toward other species, urgently needed in the accelerating loss of species driven by the climate change. Care as an important aspect of vulnerable human beings has been acknowledged as early as in Plato (1997) dialogue *Alcibiades 1* from the mid-4th century BCE,^10^ in which the capacity to “care of oneself” is needed in order to care for the city’s affairs. For Seneca, a representative of the Stoic tradition, care was “the key in becoming truly human” (Jecker and Reich, 1995, p. 350). And in the second-century Latin collection of myths, edited by Gaius Julius Hyginus, human being is created by care (*cura*) (1960). This myth was picked up by Martin Heidegger (1980/1927), who gave care (*Sorge*) the status of being the ontological structure of human beings (*Dasein)*.

It may be a surprise that care has not received the attention it deserves in psychoanalytic theorizing, although, I believe that many, if not all, would agree on its vital role in psychoanalytic practice.^11^ To some extent, other frequently used notions are used, such as “containing” and “holding,” instead of “care.”^12^ But none of these terms are synonymous with care. They are more technical and narrower than care, although they do capture significant elements in the concept “care” that I have in mind in this context. Monique Schneider has noted that there is a resistance to addressing the importance of maternal care in psychoanalytic theorizing: “Generally, psychoanalysis doesn’t want to know anything about this enveloping power because it reactivates a sort of deep feminine and maternal identification” (in Breen, 1993, p. 33). We will shortly turn to a psychoanalyst who, nevertheless, puts great emphasis on the phenomenon of care, namely Winnicott. Hollway notices that the word “care” is hardly used by psychoanalysts, with the “important exception of Winnicott” (2006, p. 16). And Joanna Kellond states that “care was an abiding and important theme in Winnicott’s work, and one that distinguishes him from other psychoanalysts” (2022, p. 3). Not to be forgotten is Erik H. Erikson, for whom care played a significant role in a person’s development (Erikson and Erikson, 1998).

To capture the character of care involved in a deep primary caregiver’s care, I take my vantage point from Anzieu’s and Winnicott’s contributions, although some other psychoanalysts and thinkers will be referred to. Two relevant situations will be covered, the first one concerns the fetus in intra-uterine life, the other, the post-natal interaction between the primary caregiver and the infant.

## The interaction between the mother and the fetus in intra-uterine life

Traditionally, the relationship between a primary caregiver and the infant begins at the child’s birth. However, before such a postnatal interaction begins, there is an intra-uterine interaction between the mother and the fetus, focused upon in both Anzieu’s and Winnicott’s thinking. Anzieu (2000, p. 347) points out that the womb functions as the first psychical container. The womb is the place where a common sensitivity is developed between the mother and the fetus. Winnicott connects the intra- and inter-uterine maternal caring with “a very special state of the mother, a psychological condition which deserves a name, such as *Primary Maternal Preoccupation*” (Winnicott 1958/1956, pp. 301–302) (italics in the original). This “primary maternal preoccupation” lasts from late pregnancy until a few weeks after the birth of the child. The character of the “primary maternal preoccupation” is more developed from the perspective of the mother in Winnicott’s writing, although he also has something to say about the perspective of the fetus in its development to an infant, in that he asserts that the fetus’ predicament prepares the infant for a certain lack of protection that it will experience after its birth, in terms of environmental impingement. If I have understood Winnicott correctly, the fetus is able to experience a withdrawal from a state of distress, and a return to a satisfactorily state where no withdrawal or reacting is needed, which he calls a state of being, and as such “is the only state in which the self can begin to be” (1958/1949a, p. 183).

Another psychoanalyst who has written on the prenatal/perinatal relationship between the fetus/infant and the mother, from both the mother’s and the fetus’ perspectives, is Lichtenberg-Ettinger (1997), who ascribes a significant philosophical, ontological meaning to this relationship. Her matrixial theory of an unconscious borderspace where the mother and the fetus co-emerge, is influenced by many psychoanalysts. In common psychoanalytic thinking, the birth of the subject usually is thought of as a post-natal event, but Lichtenberg-Ettinger places the beginning of the event in the late prenatal phase, constituted by a co-emergence between I and non-I, a subjectivizing process between partial subjects, and not between two separate subjects or a subject-object relationship. As far as I understand this “I and non-I awareness” applies to both the mother and to the fetus, although in a minimalistic way. Lichtenberg-Ettinger states: “A minimal discerning of co-emergence of *I* and *non-I* with minimal affect takes place in the feminine/prenatal *borderspace*” (italics in the original) (1997, p. 370).^13^ This co-emergence between the mother and the fetus is described as the fetus’ becoming-subject, influenced by the mother’s phantasy to be a mother, at the same time as the mother is influenced by the fetus. This co-emergence constitutes the earliest elements for fostering a possible healthy relation, not least by means of Bion’s alpha-function and K-link (Bion, 1962, 1963, 1970). The development of the fetus goes from being an object to becoming a partial subject, a subject-to-be or a matrixial subject. In the phantasy of the mother-to-be, “the fetus is not entirely ‘me’ and not totally stranger… is no longer an object but not yet a subject” (Lichtenberg-Ettinger, 1997, p. 370). This borderspace cannot be likened with Mahler’s autistic or symbiotic stage (Mahler et al., 1975). The differentiation-in-co-emergence is neither something that takes place after a symbiotic stage but implies from the very beginning a differentiation between the fetus and the mother. More precisely, the matrix is described as a borderspace “of simultaneous co-naissance, co-emergence, and co-fading of several elements in primary differentiation” (Lichtenberg-Ettinger, 1997, pp. 381–82).

Lichtenberg-Ettinger uses several concepts to describe the prenatal borderspace that are known from other psychoanalysts’ description of postnatal processes and phenomena, among others, “bodily I (Freud), originary space and pictograms (Aulagnier), unthought known (Bollas), beta- and alpha-elements, alpha- and proto-mental processes (Bion), deep psychological structures (Ogden), and subjective-objects (Winnicott)” (*Ibid.*, p. 379).

This primary prenatal matrixial phase makes up the basis for the ensuing postnatal, maternal awareness of “separateness-without-aggression” (*Ibid.*, p. 392).^14^ I have highlighted the possibility of the matrix to foster a healthy development, but a negative development cannot be ruled out due to somatic or emotional disturbances, with the risk of a pathological development.

The existence of a prenatal interaction between the infant and the biological mother is also supported by empirical, quantitative-statistical, studies, some of which have been based on John Bowlby’s attachment theory (e.g., Brandon et al., 2009; Lorensen et al., 2004; Rubin, 1975; Wilson et al., 2000). One finding is that the fetus usually becomes increasingly imagined by the mother as a human being the longer the pregnancy proceeds. By the 36th week of the pregnancy, 92 percent of the female participants thought of their fetus as a “real person,” and they become much more involved in interacting with them, by talking and stroking the fetus (Lumley, 1982). In such a way, mothers can achieve a sense of “we-ness” with the fetus (Rubin, 1975).

## The primary caregiver’s post-natal care

Anzieu’s and Winnicott’s descriptions of the post-natal interaction between the primary caregiver and the infant convey to a large extent the same idea of what is needed for the care of the infant, although differences between them also exist.

I will begin to examine these two theorists with a schematic presentation of Anzieu’s theory of the “skin-ego” (1989). His original contribution lies in his way of founding the psychological ego in biology, rather than in the brain or by means of biochemistry, which are usually referred to in this context. For Anzieu, the function of the skin is central. He claims that the importance of the skin is undervalued. Its function is taken for granted and practically not noticed until it fails. In fact, the skin is the most important sense. If more than a seventh of it is destroyed, by for example being burned, one will die. The skin has many functions, one of which is to make up the boundary to the world, thereby fulfilling a protective function to the world, it protects “our individuality” (*Ibid.*, p.3).

There is an analogy between our biological structure, the skin, and our psychic functioning, which Anzieu calls the “skin-ego.” Anzieu claims that “[i]n the same way that the skin functions as a support for the skeleton and the muscles, the Skin Ego fulfils a function of *maintaining* the psyche” (italics in the original) (*Ibid.*, p. 98). His model highly develops the relationship between the skin and the skin-ego.^15^

For my purpose, I want to underline his idea of the skin-ego as based on the biological structure of the bodily skin together with a containing and holding mother. It is the contact and interplay between the mother’s body and the infant, and the way that the mother responds to the infant’s sensations and emotions, that make it possible for the infant to have a fantasy of a common skin with the mother. The skin-ego is like a mixture of the infant’s concrete sensory experiences intermingled with fantasies. The optimal is if this fantasy about a common skin is of such a kind that it can keep the mother and the infant together, while at the same time signify their mutual separation.

The skin-ego functions as a container or a psychic envelop for psychic contents. This function is initiated primarily by the mother or the mothering environment. It is noticeable that Anzieu emphasizes his preference for the terms “mother” or “mothering environment” instead of the term “maternal,” which he states would limit the environment to the biological mother (*Ibid.*, p. 55). In his French original edition, it says “maternant” for “mothering,” and “maternel” for “maternal.”^16^ Despite Anzieu’s distancing himself from the term “maternal” due to its connotation of biological mother, he nevertheless refers to the womb as functioning as the first psychical container, where a common sensitivity is developed between the mother and fetus. It would seem that this common history between the fetus and the biological mother is not enough to justify the use of “maternal care” when describing the infant’s post-natal fantasy of a common skin with the mother.

Anzieu sees many connections between his idea of the skin-ego with different theories of other psychoanalysts, one of which is Winnicott’s “holding,” to whom I will turn now.

Winnicott has, probably more than anyone else, stressed the real, intimate and caring relationship between the mother and the infant. I will here try to give a representative account of his comprehensive production. For Winnicott, the development of the infant begins with, in his terms, “absolute dependence.” Absolute dependence entails a devoted mother’s so-called “primary maternal preoccupation,” beginning, as I stated earlier, at the end of the pregnancy and lasting for a few weeks after the birth of a child. In this stage of care, the mother has a heightened sensitivity toward the baby, to the extent that it would “be an illness were it not for the fact of pregnancy” (1958/1956, p. 302). Winnicott also establishes in this article that the mother is “the most suitable person for the care of that baby” (*Ibid.*, p. 304). She is “given over to” the care of the baby and is “very much identified with the baby and knows quite well what the baby is feeling like,” at first “it seems like a part of herself” (1965/1963, p. 85). In one of his articles, he states that the infant’s needs require a *perfect* environment. In the course of its development, a *good-enough* environment seems to suffice, allowing for a certain failure of adaptation on the part of the environment (1958/1949b, p. 245). In another article, a good-enough mother is someone who is capable of “an almost complete adaptation to her infant’s need” (1958/1951, p. 238). “Devotion” is a significant word in this context, which captures the mother’s special relationship to the infant. (1958/1951; 1965/1960a, p. 148).

No doubt, it is the biological mother or in Winnicott’s words “the infant’s own mother” who is more likely to be good enough, compared to some other person (1958/1951, p. 238). Although in the same text, he reassures us that this does “not necessarily” need to be the case (*Ibid.*, p. 237). There are occasions in which Winnicott relativizes the function of the primary caregiver: “When a woman has a strong male identification she finds this part of her mothering function most difficult to achieve, and repressed penis envy leaves but little room for primary maternal preoccupation” (1958/1956 p. 302). He allows for the possibility that the father functions as a so-called “another mother” in this very early care that concerns “the infant’s relationship to the mother” (1965/1960a, p. 142). But the father as another mother is not yet a significant male person.

A key notion in capturing the maternal care of the baby is “holding” (e.g., 1958/1949a; 1965/1960b). Holding covers a broad spectrum of aspects, physiological as well as psychological ones. To “hold” means to follow the day-to-day changes in the unique baby’s development. What is important in the beginning of the baby’s life is that the infant and the maternal caregiver form a unit. The fragility of the infant requires a mother who can perform such a holding that the baby is not ever aware of any maternal care. When the maternal care fails, the infant “becomes aware of reacting to some impingement” (1965/1960b, p. 52). In Winnicott’s words, the baby needs to experience an “illusion of omnipotence,” enabling a “continuity of being,” to begin to exist. The illusion of omnipotence is made possible due to the mother’s sensitivity to resonate with the infant’s impulses, for example sucking the breast, in such a seamless way that the infant feels it has created it. The breast is experienced as part of the infant. An alternative to such a healthy development at this early stage of life, is a baby who is forced to react to environmental impingements, meaning an environment that is not protective enough, which amounts to feelings of annihilation.

In the infant’s interaction with the mother, the mother is less visible for the infant in Winncott’s thinking than in Anzieu’s. Indeed, the ideal for Winnicott is that she does not appear at all for the infant. In Anzieu’s view, the mother is at least a part of the infant’s fantasy of a common skin. For Winnicott, her presence is completely hidden from the infant in its “illusion of omnipotence.” Her presence is not only hidden, but the “satisfactory care” that the infant receives is not ever apparent. Winnicott talks about the “[u]nawareness of satisfactory maternal care” (*Ibid.*, p. 52).

Winnicott’s description of the mother’s task can sometimes be conceived of as not only unrealistically demanding but also idealizing. However, he does enumerate in one article 17 reasons for the mother to hate her infant from the very beginning (1958/1947, p. 201).

## The meaning of the infant’s sex and associated gender ideals

A general impression when consulting psychoanalytic literature is that the issue of the infant’s sex is not reckoned with. It first becomes relevant once the infant has grown to become a child in the late preoedipal phase or in the oedipal one. If we put our focus on the infant, it is clear that the infant can hardly have any awareness of its own sexual belongingness. The child does not obtain a sense of sexual differentiation until about 18 to 30 months of age. The child’s core gender identity is, according to Robert J. Stoller, fully established between 18 to 36 months of age, “before the fully developed phallic stage” (1968, p. 30).

To my knowledge, neither Anzieu nor Winnicott discuss the significance of sexual belongingness of the infant in the very early interaction between the primary caregiver and the infant. Although at the time of the first Not-Me possession, that is when Winnicott states that the use of transitional objects begins, at about 4 to 12 months of age, Winnicott lets us know that “[i]t is important to note… that there is no noticeable difference between boy and girl in their use of the original Not-Me possession” (1958/1951, p. 232).

Even if the care of the infant is first and foremost a question of fostering a healthy ego-development, we can assume that the primary caregiver and other people, consciously and unconsciously, ascribe and project sex/gender messages to the infant. The primary caregiver’s understanding of the infant’s sexual belongingness is immediately permeated with gender meanings, such as wishes, fears, norms, ideals and ideas of what it means to be a girl/woman or a boy/man. This begins as soon as the primary caregiver knows the sexual belongingness, regardless of whether this happens before the child’s birth or in connection with its birth.

Empirical research has shown that parents already in the first hour after the birth of their child handle boys and girls differently. As for boys, they are activated, while girls are left in peace. The way of speaking to the boy and the girl is also different. The girl is given epithets like “little heart,” “sweet” and her body is soft and nice. The boy is attributed as having “big hands” and “muscles on his arms,” and he is asked “are you thinking of running away already” (see Carlberg, 1994).

Judy Shuttleworth (1997, p. 48) points out that infants under six months are especially sensitive to the styles of their care. The infant becomes familiar with a certain style of caring from their primary caregiver which gives the infant a sense of continuity in the caring.^17^ It would not be controversial to claim that the caring style depends on both the sex of the infant and the sexual belongingness and gender ideals of the primary caregiver. And even if the infant is too young to give any gendered meaning to the caring style, it may later bestow it with gendered meaning, in line with Freud’s idea about deferred action (*Nachträglichkeit).*^18^

Even though the interaction between the primary caregiver and the infant involves a sex/gender dimension, the interaction should not be reduced to sex/gender. Indeed, for the infant/child there is a distinction between I and the other preceding any differentiation based on sexual belongingness. From the perspective of the primary caregiver, one can assume that the care of the infant vacillates between the involvement of a sex/gender dimension and an I-You relationship as described by Buber.^19^ The You for the primary caregiver correlates to the infant’s nascent ego or I.

This uniqueness of every human being is important and helps us to liberate ourselves from a categorizing attitude, which Buber’s I-You philosophy draws upon. Buber (1970/1923) asserts that the basic relationship between human beings is an I-You relationship. The relationship between I and You is unmediated, whereas the word pair I-It depicts something mediated and remote from You. When I turn to a human being as my You, Buber lets us know that this human being is “no longer He or She, limited by other Hes and Shes” (*Ibid.*, p. 5). Winnicott’s description of holding in terms of following the unique baby’s development is relevant here.

I have touched upon this I-You dimension earlier on, when referring to Alizade (2010) complaint that psychoanalysis has not dealt in sufficient depth with the fact that we are first and foremost human, before having been classified sexually, and she states that “[t]he binary division laid down by social rules does not eliminate the human character of the species” (*Ibid.*, p. 203). This “human dimension” functions as a secure base, a feeling of security and well-being, which has more to do with the Freudian life drive, and I would add the self-preservation drive, rather than the death drive, and I would add the sexual drive/libido. One function of the skin-ego that Anzieu (1989) emphasizes is to support sexual excitation; if the skin-ego is not sufficiently containing, sexuality can be too traumatic. Anzieu’s observation points to the significance of the first challenge and achievement in life, namely feeling and affirming one’s existence. The skin-ego serves the function of shaping and affirming one’s existence. Whereas, sexuality is dissolving; in Freud’s meta-psychological language it partakes of a discharge/diminution of the quantity of excitation (Freud, 1920; Schaffer, 2010). The ego can be seen as the source of existence, and without having a sense of oneself being alive, the capacity of (libidinal) wishing and desiring will be hampered (Karlsson, 2004, 2010, ch. 4).

In line with Anzieu’s thinking, Alizade states that “[t]he functions of the skin-ego are eminently at the service of the life instinct” (1999, p. 24).^20^ A development of “enveloping oneself” or “being one’s own person” can be discerned. Alizade (1999, p. 29) describes a kind of “instinctual dynamics” in terms of three periods, which can be normal or pathological. The first period begins at birth, one of being enveloped. One’s body is felt and vibrated (*senti)*, enwrapping the infant, combining with a “bath of voice” or “bath of prosody,” in particular from the mother. The next period is when “one is enveloped by oneself,” which consists of an internalization of the first period, in that the function of being enveloped becomes one’s own. The third period is about “enveloping another,” loving and caring for others. This is also a description of a development from a pre-sexual world, “outside the sex-gender system and anything connected to it,” which subsequently becomes interwoven with the sexual world, but still preserving its autonomy of existence (2010, p. 204).

## Discussion and concluding remarks

I want to wrap up my article in terms of some assertions which follow from the presentation in the article:

1. My first point is to refute an essentialist stance which would mean that the biological mother is, on the basis of her biological constitution, necessarily the most fit person for caring for the infant. The mother is forced to have a certain intimacy with the fetus, but that fact in itself does not necessarily imply any specific psychological meaning. For some, it is beneficial for the future relationship with the infant/child, for others it may be an obstacle. The refutation of biologism or biological essentialism is implied in the distinction made between sex and gender; there is no way to draw any necessary (gender) characteristic from a person’s specific sex. Furthermore, in line with a fundamental psychoanalytic condition, one can never draw any conclusion about meaning from objective facts. In psychoanalysis, the question is always about how one relates to facts; uncovering the meaning with which the facts are imbued.
2. My second point concerns the conceptualization of the relationship between the primary caregiver and the infant. One important task is to examine the influence of sexed/gendered language on our thinking and, if needed, try to develop a more adequate way of understanding and describing the interaction between the infant and the primary caregiver (cf., Taipale, in press). Obviously, it is an impossible task to find a language which would reflect the infant’s experiences in any exact way. Nevertheless, we should try to understand the infant’s experiences and describe them in as appropriate words as possible. There is no possibility for the infant to be aware of a whole integrated person, much less the sexual belongingness of the primary caregiver. Even if the denominations of “mother” and “father” is meant symbolically, they can easily be taken in a more realistic sense.
  A good reason to avoid uncalled-for sexed/gendered expressions is to resist stereotyped, and at worst, essentialized ideas. Another benefit may be that it facilitates an open-minded attitude, free from conventional thinking or taken-for-granted circumstances, possibly leading to a deepened understanding of the caring process. Having said this, I do not think that we will be able to free ourselves completely from an understanding of the early primary caretaking, in which sex and gender would be of no interest. And in the course of the child’s development, the identity of belonging to a specific sex also entails a process of disillusionment in that the omnipotent wishes to be everything are to be rejected in favor of an acceptance of reality.
  In addition to the distinction between sex and gender, the notion of “ego-identity” could be fruitful in this field of research. The formation of human identity precedes and is irreducible to the sex and gender distinctions. To reiterate Alizade’s words: “In the beginning, before having been classified as male or female, a human being is born” (2010, p. 203). The development of an ego-identity is best secured in an attitude of an I-You-relationship, the most open and intimate encounter between the primary caregiver and the infant.
  From what has been said thus far, I would like to see research investigating the primary caregiver’s interaction with the infant, comprising the sex/gender and the I-You dimension. I find these to be neglected areas of research in psychoanalytic thinking and theorizing. Besides, Susan McNamara et al. (2025) point out the lack of literature on the experience of gay, lesbian and queer parents, neglecting the important tasks in caregiving, at the cost of reifying these tasks along gender lines (p. 865).
3. My third point concerns gender ideals. The distinction between sex and gender should be kept in mind, where sex (female, male, non-binary sexual identity) is experienced as something given and non-acquired, whereas gender (femininity, masculinity) has to do with ideals, involving actions, behaviors, character and psychological and cultural levels of meaning. It is well known that in psychoanalysis, to the extent that care is brought to fore, it is linked to feminine traits and ideals, the most well-known contribution is probably *The reproduction of mothering*, by Chodorow (1999/1978). Jane Flax points out that the pre-oedipal period is really the period where the significance of the female sex receives some acknowledgement by many psychoanalysts, both for the mother and for the little girl’s personality development, but not for the boy, whose personality development is located to the oedipal period. This “masculinist bias” found in Freud “recurs in the work of many subsequent analysts, both ‘orthodox’ and Lacanian” (1990, p. 79).^21^
  If we move outside the sphere of psychoanalysis, the connection between care and femininity has even developed into a feministic ethic of care, as opposed to a masculine ethic based on a Kantian ethic, guided by autonomy, abstract thinking and rationality (cf., Gilligan, 1982; Tronto, 1993).
  Let us turn to the masculine gender, more precisely phallic masculine ideals.^22^ Even if it seems that both Anzieu and Winnicott assume that the mother is the most suitable person to be the primary caregiver, Winnicott, nevertheles, points out that the primary maternal preoccupation is difficult to achieve for a woman with a strong male identification (1958/1956 p. 302). Furthermore, they both open up for the possibility that someone else could carry out this function. For example, the father as another mother, is mentioned by Winnicott (1965/1960a), p. 142).
  This brings me to addressing “phallic masculinity” as a gender category, which cannot be reduced to the male sex, although it allows itself to be easier adopted by boys/men than by girls/women, and perhaps by people who identify themselves as non-binary, due to both psychological, developmental experiences and cultural norms and ideals.^23^
  Let us establish that the gender ideals, implied in phallic masculinity, are coveted by many men, and even many women, even though they do not usually identify themselves with such ideals. In the case that someone, most often men, strives for a phallic identity, we may denote such a striving as a “project” – the phallic masculine project. A project consists of possibilities that are not yet realized, as opposed to “identity” which describes an existing state of being. Elsewhere, I have argued that phallic masculinity as a project is doomed to fail, since it implies a rejection of existential human conditions such as vulnerability and helplessness, as well as those phenomena aimed at soothing these conditions, such as (maternal) caring (Karlsson, 2023). These two factors: the importance for the man to be a strong, masculine man and the unattainable project of achieving something like a masculine identity may explain the recurring discussions about men being in crisis.^24^ In other words, phallic masculinity can be conceived of as a reaction to fundamental human predicaments, rather than a natural process of development. This kind of reaction, or maybe better expressed as a “counter-acting,” plays out later in the pre-oedipal phase, and particularly in the oedipal phase, but in the worst-case scenario, it can continue throughout life. It is a kind of emotional rebellion to an initial identification with maternal care.
  Included in the phallic project is a polarization between the sexes and between masculinity and femininity.^25^ The capacity to achieve care becomes greater insofar as one is capable of affirming identifications with maternal care/the care of the primary caregiver. This may be a great challenge, to emotionally integrate values and traits that hitherto have been repudiated by many men/fathers (cf., Diamond, 2004, 2013, 2021; Fogel, 1996, 2006; Kaftal, 1991). Thierry Bokanowski (2010) makes the observation that men who show feminine tendencies feel great shame, which contrasts starkly to women who have no problems coping with possible phallic and masculine tendencies. This difference in emotional reaction makes Bokanowski wonder if men’s exclusively negative attitude to femininity is due to the feminine dimension being too intimately interconnected with maternal identification.
  However, the concept “care” does not only connote emotional capacities, but can also involve hands-on interventions and practical actions, which would resonate better with a phallic masculine character. So, no doubt, there exists sacrifice and self-less generosity even amongst men.
  Nevertheless, when it comes to the qualities of an emotional caring relationship, as is called for in the care of an infant, phallic masculinity is not equipped to handle such situations. There are certain traits in holding, containing, projective identification, and the fantasy of a common skin between infant and the primary caregiver, which would be difficult for phallic masculinity. For example, the receptive attitude of the primary caregiver involves a certain passivity in order to digest painful evacuated emotions from the infant; an attitude that is opposite to the preferred phallic attitude of initiating actions. Here, one must simply endure the state of being rather than making attempts to transcend it through actions, that is, trying to move away from it. It requires a form of dwelling on feelings rather than exercising an instrumental attitude to solve problems by action and doing. Winnicott (1991/1971) makes a distinction between, on the one hand “female elements” referring to a capacity to be or being, and on the other “male elements” which have to do with a capacity to do. The capacity to be precedes the capacity to do. We are also reminded of the importance of the primary caregiver of holding oneself back, even to the extent that it enables the infant’s “illusion of omnipotence.”
  Another significant aspect in emotional caring is the experience of mutual sharing, a quality which Anzieu emphasized with his “fantasy of a common skin,” also visible in projective identification. The phallic masculine project, cultivated in and producing an atmosphere of competition and rivalry, demands sovereignty and self-sufficiency. An intimacy in form of a mutual sharing can motivate strong resistance and disgust. A good example of this is when the psychoanalyst symbolizes the motherly containing function, creating deep bodily discomfort. It can often be experienced as something sticky, viscous, and stifling.
  These bodily reactions call for a comment concerning sensory modalities. The early caring of an infant is a story about touching each other, skin to skin, as in Ogden’s “autistic-contiguous position”; skin being the most important sense, according to Anzieu. And in his idea of a fantasy of a common skin between the mother and the infant, the tactile sense and the skin are predominant, in contrast to the phallic phase with the penis/phallus as organ in focus in its visible character. The tactile sense is better equipped to capture intimacy between people, in comparison with the visual sense, which has a more objectifying and distancing character. Even if the male identity processes begin before the phallic phase, the penis/phallus, nevertheless, becomes the central marker for being a male, and calls forth associations to the masculine. We live in a culture and a (natural) scientific climate which prioritizes the ocular sense, and, at least to some extent, this is also the case for psychoanalytic theorizing as well. A correspondence between a phallic bias and ocular centrism can be discerned.
4. My fourth and final remark concerns the position of the biological mother as the primary caregiver. Not to be forgotten, it is the quality of the caring that matters and not the sexual belongingness of the caregiver. However, the biological mother, everything else being equal, might have a certain advantage in carrying out a beneficial early interaction with the infant. The biological mother has a unique position in that she carries the fetus for a long time in her own body. Already before the birth of the child, there is an interaction going on between the mother and the fetus/infant. Other people, for example the father, can also have a kind of pre-natal relationship with the fetus, but it is not in the same bodily and concrete way as it is for the biological mother. We are reminded about Winnicott’s “primordial maternal preoccupation” which begins in late pregnancy and lasts a few weeks after the birth of the child. Anzieu expresses a similar idea, in that the biological mother’s womb is the place where a common sensitivity is developed for the mother and the fetus.
  Finally, I want to refer to Lichtenberg-Ettinger’s matrixial theory, which also entails a feminist plea against a phallic logic. A phallic logic is a logic which defines the woman as lacking something that the male possesses; from the beginning everyone is a “small man,” the penis is the only existing genital organ. Such a phallocentric logic highlights the presence or absence of a penis/phallos but neglects other bodily differences between the sexes.^26^

However, “The Matrix is not the opposite of the Phallus; it is rather a supplementary perspective. It grants a different meaning” (Lichtenberg-Ettinger, 1996, p. 125). Lichtenberg-Ettinger’s unconscious matrixial perspective, illuminating the female bodily opens up a shared matrixial borderspace, for both the female fetus as well as the male fetus, although it is especially for women that it becomes a “non-phallic sex-difference issue” (1997, p. 397). Thus, in a certain sense both females and males have the same origin, although the females have potentially a double experience, in the sense that is not limited to the early prenatal life in the mother’s womb, but also a possible retroactive sense of phantasies created out of her prenatal life. To quote Lichtenberg-Ettinger (*Ibid*, p. 374):

I suggest that we examine the potentiality of the feminine/prenatal *encounter* as a sex-difference issue mainly for women, a beyond-the-phallus measure of difference that is not opposite from nor the same as nor symmetrical to the phallic difference for men. (Italics in the original).

At the wake of becoming a mother, the woman may connect her prenatal sexual belongingness to the present life conditions, which can be understood as a kind of *nachträglich* connection; “[t]he matrix is a sex-difference that ‘remembers’ the female body….” (*Ibid.*, p. 368) Something hidden in the unconscious, with roots in the prenatal life, receives meaning in the presence of a certain situation. For males, the situation is thus somewhat different. Males tend to repress or foreclose these prenatal traces; they become absent rather than making up a creative connection as can be the case for women. In other words, the structure of matrixial borderspace may pose a narcissistic threat to males as its existence belongs mainly to their past and not to a potential future. Insofar, as it becomes presence, Lichtenberg-Ettinger’s claim is that then it, first of all, has to do with deep regression and disintegration, in stark contrast to the case for women where such a regressive state also is creative.

Thus, in certain respects, the biological mother is acquainted with the infant from its prenatal life, where a nascent relationship between the biological mother and the infant is taking place. Furthermore, if the biological mother is able and willing to breastfeed the infant, this may allow for a unique possibility of a close, intimate relationship with the infant. In other words, the biological mother has certain potentialities that no one else has, and these can have advantageous effects on the early interaction with the infant. However, a dialectic thinking is called for; the outcome of the intimate mother-infant relationship can foster a rewarding and developing relationship, although this unique intimacy can also cause difficulties and obstacles in forming a mother-infant unity.^27^ Perhaps one can formulate the mother’s unique position in fostering a beneficial interaction with her infant, as a privileged position. This should not, however, lead to essentialist thinking and categorizations of persons from a purely biological perspective.

## Data Availability

The original contributions presented in the study are included in the article/supplementary material, further inquiries can be directed to the corresponding author.

## Footnotes

1 Psychoanalysis has been accused of treating the issue of transgender in a disrespectful way, which hopefully belongs to the past. Examples of recent psychoanalytic contributions to the theme of transsexuality/transgender are: Bell (2020), Blass et al. (2021), Drescher (2020), Lemma (2012, 2013), Gullestad (2024), Saketopoulou (2020) and Schei Jessen et al. (2025).

2 Wendy Hollway deplores that the distinction between sexual and gender differences “has been lost with the dominance of a social constructionist paradigm on identity that claims that all differences are socially produced and therefore gender differences” ((2006, p. 36). The psychoanalyst Gregorion Kohon asserts that “the complexity of gender… cannot be reduced either to a social construction or to biological differences” (in Lemma, 2018, p. 1103). For a critical psychoanalytic clinical analysis of poststructural theories, see Lynne Layton (2004). Other relevant psychoanalytic critical viewpoints of social constructionism are, for example, James Hansell (2011) and Rosemary M. Balsam (2012). See the sociologist Henry Rubin (1998) for a phenomenological critique of a poststructuralist view on transsexual persons’ lived experiences.

3 An important distinction to make is between sex/sexual identity and sexuality, something that is easily overlooked in English. Elizabeth Grosz states: “In my understanding, the term ‘sex’ refers, not to sexual impulses, desires, wishes, hopes, bodies, pleasures, behaviors and practices: this I refer to the term ‘sexuality’. ‘Sex’ refers to the domain of sexual difference, to the question of *morphologies of bodies*” (italics in the original) (1995, p. 213).

4 Apparently, psychoanalysis is no exception in struggling with an unclear terminology concerning sex and gender. For example, Michael J. Diamond asserts that “the landscape of psychoanalysis and gender abounds with conceptual, terminological, technical, and socio-political difficulties” (2013, p. 2). Emilce Dio Bleichmar claims that even if there is plenty written about gender in psychoanalysis during the last decades, still today “neither its conceptual aspect nor its clinical application is clear” (2010, p. 177). And Rosemary H. Balsam states: “Contemporaneously, though, in spite of socially influenced improvements at gross level, we are quite stalled in psychoanalytic theory-building regarding sex and gender. Our theories lack cohesion in this arena, and lag very much behind our clinical dealings and *potential* for new clinical explorations, which are thus understudied, underreported, and therefore under-theorized” (italics in the original) (2019, p. 13). For a discussion about why it has been so difficult to determine the meaning of sex and gender (see Karlsson, 2023, ch. 1).

5 I find it confusing that “gender” is used many times in the literature when the term “sex” would be more appropriate. The nouns used for sex should be female/woman, male/man and intersex, and for gender the applicable terms are feminine and masculine. However, I have here chosen to comply with referred authors’ terminology which means that their use of “sex” and “gender” may not be in line with mine.

6 It should be noted that all kinds of identities are not to be conceived of as given, as non-acquired. A distinction can be made between a non-acquired identity, such as I said, one’s sexual belongingness, and an acquired identity, such as for example one’s professional identity. The non-acquired identity can possibly be affirmed or experienced as irrelevant to one’s being, but it can hardly be changed, developed or dropped by means of acting or behaving in a certain manner. An acquired identity, such as a professional identity, for example identifying oneself as a psychoanalyst, does change the individual through actions and perseverance to acquiring such a profession.

7 Here, one can also refer to Paul Ricoeur’s (1992) discussion about the identity in terms of *ipse* (identity as self-hood) and *idem* (identity as sameness), where *ipse* concerns a self-consistency that is not dependent on some unchanging core of the personality. By means of *ipse*, continuity can be made comprehensible in spite of change, variation and discontinuity on the side of *idem*. These two identity limits sometimes more or less overlap one another, but *ipse* can also appear without the aid or support of *idem*. They can be said to answer different questions: *idem*/the same answers the question “what are you?”, whereas *ipse*/the self answers the question “who are you?”.

8 As said, my terminology is different from many others within this field, since I ascribe identity to sex. When it comes to transgender, Avgi Saketopoulou states that trans “is a still-evolving term,” which currently “describes a heterogeneous set of individuals who share a felt sense of misalignment between experienced gender and the gender to which they were assigned at birth on the basis of observed sex” (2020, p. 1019). In my understanding, the assignment “at birth on the basis of observed sex” entails a claim of identity, something that the baby is. Sex is not simply the observation of embodied material, but an assignment of sexual identity. This original assignment, followed by others’ third person perspective assignments and intersubjective processes, which naturally can be cohesive or disjointed/divided with respect to the baby’s/child’s sexual identity, make up the conditions on which the baby/the child struggles to find out what its sexual identity is. This eventually becoming an issue of sexual (cohesive or disjointed) self-identity. Implied in one’s sexual identity is the question of what is expected from this identity, and eventually the potential liberating possibility of how to respond to social and cultural expectations upon this sexual identity. Thus, what is implied in my distinction between sex and gender, is that the sexual identity opens up possibilities, without prescribing any determined gendered meaning; a conceptualization which actualizes the value of authenticity within this field of research.

9 Virginia Goldner (1991, p. 249) asserts that gender identity reflects a normative ideal which psychoanalysis has absorbed uncritically. She conceives of gender as a necessary fiction. Another feminist, who is not a psychoanalyst, critical of the use of the gender concept is Toril Moi. She maintains, in referring to Simone de Beauvoir and Gayle Rubin, that that the use of “feminine” and “masculine” as descriptive positive terms risks fostering sexual based stereo-types. Her suggestion is “to stop thinking in terms of gender altogether. To me, that means trying to produce a society without sexist ideology or gender norms, without oppressive myths of masculinity and femininity” (2005, p. 112).

10 It is uncertain when this dialogue was written and it is quite likely that it was composed by Plato’s disciples after his death in 347 BCE.

11 One might wonder why the concept of ‘care’ has not achieved a more prominent status within psychoanalytic thinking. Could it be that it seems to be too mundane for a theoretical concept, compared to the task of creating a language suitable for elusive unconscious processes? No matter what the reason, it may be worthwhile to use a language which resonates with our lived experiences and everyday language. Bruno Bettelheim (1983) has testified, in a thought-provoking way, how Freud’s everyday emotional language lost its human and personal meaning for him once he encountered the English translation with its abstract, scientific style. For example, the German “Seele” was translated to “mind,” and “Das Ich” and “Das Es” to the Latinized “Ego” and “Id.”

12 See Thomas H. Ogden (2004) for a discussion of the relationship between Winnicott’s holding and Bion’s container-contained model. These concepts are often confused with each other but should be differentiated: “Winnicott’s holding and Bion’s container-contained represent different analytic vertices from which to view the same analytic experience. Holding is concerned primarily with being and its relationship to time; the container-contained is centrally concerned with the processing (dreaming) of thoughts derived from lived emotional experience” (p. 1362).

13 Lichtenberg-Ettinger’s innovative work on subjectivity as an encounter at a shared borderspace has been applied to other fields other than the prenatal/perinatal encounter between the fetus/infant and the mother, such as trauma and esthetics. Thus, her theorizing has much wider implications than being a description of this earliest encounter taking place in the womb (cf. Pollock, 2006).

14 Based on empirical data concerning the experience of becoming a mother, Hollway (2011) establishes that the mothers’ descriptions had to be understood as a continuity of prenatal, perinatal and postnatal experience. Feeling another’s life inside one’s body was a weird, ineffable experience. To do justice to the experience of becoming a mother, Hollway was helped by taking into account Lichtenberg-Ettinger’s matrixial model.

15 Alizade (1999), whose influence from Anzieu is explicitly recognized, describes the development of the skin-ego from the infant’s birth in the following way: “The mother’s body becomes the main covering and fulfills externally the enveloping function that biology meant it to provide from within. From ‘one body for two’ it is transformed into ‘one skin for two’, the somatic skin will have to be constructed at the psychic level in the gradual constitution of a skin-ego” (p. 26). Lichtenberg-Ettinger (2006) points out that the “intrauterine contact brings forth the object of touch” (p. 66). Another psychoanalyst to be mentioned in this context is Ogden (1992) who argues for the existence of a primitive “autistic-contiguous position,” which constitutes a rudimentary “I-ness.” This position concerns a sensory-dominated, pre-symbolic generation of experience. The term “autistic” refers to a “normal autism,” in terms of isolation and disconnectedness, and “contiguous” refers to sensory connections, such as the touching between the mother and the infant, skin to skin.

16 In the original French edition, Anzieu prefers the term “maternant” rather than “maternel.” He writes (1985, p. 54): “Afin d’étudier le plus précocement et le plus systématiquement possible la dyade nourrisson-entourage (que je préfère appeler maternant plutôt que maternel pour ne pas limiter l’entourage à la mère biologique)…”.

17 We can imagine that the infant is handled in such a way that the occasions of care are bound together by a structural commonality or unity. This holding (Winnicott) or fantasy of a common skin (Anzieu) is manifested in, for example, the primary caregiver’s and the infant’s mutual touching, the caregiver’s lap as a containing boundary and protecting ground and the bodily warmth and softness which the infant is held in and spoken to. The stylistic unity of care can be said to represent a global way of being, like an atmosphere.

18 Jean Laplanche and Jean-Bertrand Pontalis define “Nachträlichkeit” as follows: “Term frequently used by Freud in connection with his view of psychical temporality and causality: experiences, impressions and memory-traces may be revised at a later date to fit in with fresh experiences or with the attainment of a new stage of development. They may in that event be endowed not only with a new meaning but also with psychical effectiveness” (1985, p. 111).

19 The title of Buber’s original German edition of the book *I and Thou* is *Ich und Du*. In Walter Kaufmann’s translation of this work, he has translated Du in the Ich-Du relationship to You and not Thou. This translation is not only closer to the German original but also connotes something, in Kaufmann’s words “spontaneous and unpretentious, remote from formality, pomp and dignity” as opposed to the high-flown Thou (1970, p. 14). I comply with Kaufmann’s translation in referring to Buber’s work.

20 Alizade translates the German concept “Trieb” into “instinct” (in accordance with James Strachey’s translation of Freud’s Collected works, and which is also often the case in English psychoanalytic texts). I prefer Freud’s concept “Trieb” to be translated into “drive,” since it concerns psychical processes. However, when describing Alizade’s thinking, I comply to her terminological choice.

21 Under the next point, I want to draw attention to something that I would describe as a kind of philosophical anthropologic argument for a feminist logic, prior to a phallic logic, by referring to Lichtenberg-Ettinger’s matrixial borderspace. I chose not to present it under this point but save it for the next since it mainly concerns the conditions of the female body/the mother rather than about gender.

22 Only a few characteristics of phallic masculinity will be paid attention to. Phallic masculinity is the form of masculinity that usually is referred to when speaking about a masculine character or masculine ideals. Phallic masculinity is a coveted ideal for many and in society at large. Whereas, another form of masculinity, which, however, cannot be discussed here, called “hypermasculinity” or “toxic masculinity,” characterized by, for example, violence, sexism, xenophobia, religious terrorism, awake denunciation with most people and in society at large. In brief, hypermasculinity/toxic masculinity must intra-psychically be understood in a different way than phallic masculinity (cf., Chodorow, 2012b; Figlio, 2024; Stein, 2010).

23 It is beyond the scope of this article to discuss the trajectory of establishing a phallic masculine project. It includes developmental aspects beyond the care of the infant, which are, in particular, formed later in the pre-oedipal phase and in the oedipal phase (for some recent contributions, see Diamond, 2021; Figlio, 2024; Karlsson, 2023; Petrucelli et al., 2023).

24 David Tjeder (1999) questions whether there has been any period in history without concern for masculinity: “Men were always assumed to have been more masculine before” (p. 179).

25 Dana Birksted-Breen (1996) asserts that the other side of the boy’s denial of motherly identification is an identification with the phallus, rather than the penis symbolizing a link to someone, making a non-phallic masculinity possible. Penis-as-link recognizes lack and the need of the other, whereas an identification with phallus implies an incompatibility between masculinity and femininity, with repercussions on a societal level: “It is this phantasy of penis as phallus which confers to masculinity its coveted position, and if we think of this phantasy as ubiquitous, it goes some way toward explaining social phenomena which have given men greater power and status” (p. 651). Likewise, Karl Figlio (2010, 2024) has worked out a non-phallic masculinity, a so-called “seminal masculinity,” constructed upon the anatomy of the male. Here, it is not the possession of a penis that matters, but the man’s inner world, in terms of an inner genital phase. I have discussed a form of non-phallic masculinity in terms of “demasculinized masculinity” (Karlsson, 2023, chs. 3–4).

26 For a discussion and refutation of feminist critique in asserting Winnicott’s ideas as biologism, such as, for example, his “primary maternal preoccupation,” Hollway addresses this kind of criticism by warning for the risk of reproducing phallic logic, and she writes with reference to Lichtenberg-Ettinger’s matrixial theory: “Feminists risk reproducing phallic logic if we dismiss on ideological grounds, and thereby pathologies, this radically other state characteristic of the peri-natal period that many women experience when they become mothers” (2011, p. 20).

27 A recent study found that maternal exposure to multiple adverse childhood experiences – for example, physical abuse, emotional abuse, sexual abuse, witnessing domestic violence – was associated with increased adverse rates of perinatal outcomes (Troëng et al., 2025).
